# Farmers’ perception and adaptation practice to climate variability and change: a case study of the Vea catchment in Ghana

**DOI:** 10.1186/s40064-016-2433-9

**Published:** 2016-06-22

**Authors:** Andrew Manoba Limantol, Bruce Edward Keith, Bismark Atiayure Azabre, Bernd Lennartz

**Affiliations:** Graduate Research Program (GRP) Climate Change and Water Resources, West African Science Service Center on Climate Change and Adapted Land Use (WASCAL), University of Abomey-Calavi, Cotonou, Benin; Department of Systems Engineering, Center for Nation Reconstruction and Capacity Development, United States Military Academy, West Point, NY USA; Department of Planning and Management, University of Development Studies, Wa Campus, Wa, Ghana; Agricultural and Environmental Sciences, University of Rostock, Rostock, Germany

**Keywords:** Climate change, Farmers’ perception, Adaptations, Barriers, Vea catchment

## Abstract

**Background:**

Rain-fed agriculture remains the source of employment for a majority of Ghana’s population, particularly in northern Ghana where annual rainfall is low. The purpose of this study is to examine farmers’ perceptions and adaptation practices to climate change and variability in accordance with actual recorded weather data of the Vea catchment in Upper East Region of northern Ghana during the time interval from 1972 to 2012.

**Methods:**

Climatic data over 41-years (1972–2012) from four stations in vicinity of the catchment was evaluated to identify actual weather outcomes. A survey questionnaire targeting farmers with at least 30-years of farming experience in the area was administered in six of the eleven agricultural enumeration areas in the catchment covering 305 km^2^. Of the 466 farmers interviewed, 79 % utilized rain-fed practices while 21 % utilized some form of irrigation.

**Results:**

Results indicate that nearly 90 % of the farmers interviewed believe that temperature increased over the past 30-years, while over 94 % of the farmers believe that amount of rainfall, duration, intensity and rainy days has decreased. Nearly 96 % of the farmers believe that their farms are extremely vulnerable to decreased rainfall, droughts and changed timing of rainfall events. Climatic data of the catchment indicates a rising trend in temperature but no long-term changes in annual and monthly rainfall, thereby possibly increasing levels of evapotranspiration. While no statistical differences were found between rain-fed and irrigation agricultural types regarding receipt of external support, their approaches to climatic change adaptation do differ. Patently, 94 and 90 % of farmers relying on rain-fed and irrigation strategies respectively receive some form of support, primarily via extension services. Farmers using rain-fed practices adjust to climate variability by varying crop types via rotation without fertilizer while farmers employing irrigation practices are more likely to offset climate variability with a greater use of fertilizer application.

**Conclusion:**

The Vea catchment faces rising temperature and evapotranspiration trends. Farmers are aware of these climatic changes and are adapting strategies to cope with the effects but require support. Adequate extension services and irrigation facilities are needed to assist farmers in order to sustain their livelihoods on the long run.

## Introduction

Climate change is likely to adversely affect the lives of poor and rural African farmers, potentially undermining food security and socio-economic development if no appropriate measures are taken (Abeygunawardena et al. 2003; Adger et al. 2007; Gbetibouo 2009; Ringler et al. 2011). Adaptation to climate change impacts on agriculture has therefore become a major concern to various stakeholders in sub-Saharan Africa (SSA), with special emphasis on how to assist farmers in improving their adaptive capacity (Temidayo 2011). For any effective adaptation policy, the decisions and strategies in addressing the impact of climate change on farmers must take into account farmers’ knowledge and perception of climate change, their potential adaptation measures, and possible barriers and constraints to such adaptation (Fosu-Mensah et al. 2012).

The potential impacts of climate change on rain-fed agriculture strategies versus irrigated systems are not well understood (FAO 2007). In Ghana, for example, climate change is projected to have serious socio-economic impacts on rural farmers whose livelihoods depend largely on rainfall (Abeygunawardena et al. 2003; Fosu-Mensah et al. 2012). Yet, the extent to which such adverse effects are now and will be felt by farmers in this area depends largely on the adaptation strategies they employ in response to climate change (Gbetibouo 2009). The Vea catchment in the Upper Eastern Region (UER) of Ghana serves as the location of the Vea irrigation dam, one of the two major irrigation dams (Vea and Tono) in Ghana’s UER. The catchment area is home to many rural farming communities and is one of the major food production areas of both the region and Ghana. While the dam offers farmers the opportunity for continuous agricultural production throughout the year (Badmos et al. 2014), knowledge about farmers’ perceptions on climate change, their adaptation strategies, and barriers to adaptation has not been effectively documented (see e.g. Nakuja et al. 2012; Kanlisi and Arkum 2013).

The purpose of this study is to examine farmers’ perceptions and adaptation practices to climate change and variability in accordance with actual recorded weather data of the Vea catchment in UER of northern Ghana during the time interval from 1972 to 2012. In this study, we conduct an intensive reconnaissance field survey of the catchment area to document geographic and physical characteristics of the area as well as the various farming systems and adaptation measures practiced. A structured household questionnaire is employed to gather information on the area’s demographic farming profile, extant farming systems, farmers’ concerns for and perceptions of climate change, and the adaptation practices undertaken to build resilience to climate change outcomes.

## Background

Globally, 85 % of all farms are operated by smallholder farmers (holders of <2 ha of farm plots) (Nagayets 2005), with the population of smallholder farmers representing about 50 and 75 % of people living with hunger globally and in Africa respectively (Sanchez and Swaminathan 2005). Since livelihoods of these farmers depend directly on agriculture, they are often vulnerable to unexpected shock events that result in crop failure and food/income insecurity due to constrained resources and an inability to respond quickly to environmental changes (Hertel and Rosch 2010; McDowell and Hess 2012; cited in: Harvey et al. 2014). Patently, because the fate of these smallholder farmers will determine the outcome of the global fight in reducing poverty and hunger (Harvey et al. 2014), understanding their contextual situations and response to climatic shock events is a critical step in addressing the challenges of food insecurity.

Agricultural production and the livelihoods of smallholder farmers across Africa is particularly constrained by numerous challenges that exacerbate food security, such as disease and pest invasions, post-harvest losses, market shocks and lack of capital/credit, among others (Morton 2007). Climate change is projected to further worsen the plight of smallholder farmers as suggested by recent studies that surmise the production of main cereal crops by smallholder farmers, such as maize, rice and wheat will be negatively affected by even modest increases in temperatures (Morton 2007). Tropical countries like those in Africa with already high populations of poor and smallholder farmers are the areas expected to be hardest hit by climate change impacts (Hertel and Rosch 2010). The drought conditions in Sahara and Sahel, in particular, will worsen as rainfall is expected to decline (Ofori-Sarpong 2001). Owing to the projections of worsening climatic conditions, it is imperative that efforts be directed toward helping these smallholder farmers identify effective adaptation production systems in building strong resilience to climate change (Harvey et al. 2014).

Agriculture has long been a major pillar to Ghana’s economy, employing about 55 % of the population, contributing about 35 % to Ghana’s gross domestic product (GDP), and generating about 30–40 % of the country’s foreign exchange earnings (Fosu-Mensah et al. 2012). Contribution of the crop production sub-sector to Ghana’s agricultural GDP is about 66.2 % (MOFA 2011), while ‘agriculture-related manufacturing, such as food, cocoa and wood processing, accounts for more than 60 % of Ghana’s manufacturing industry’ (Diao 2010). Needless to say, a dramatic decline in crop production throughout Ghana attributable to climatic shock events would significantly reduce country capacity.

In spite of the crucial role it plays in the national economy, agriculture in Ghana is saddled with serious challenges that include the negative impacts of climate change (droughts and floods) as well as poor basic infrastructure (road and irrigation); socio‐cultural factors and agricultural‐related services (UNDP 2010). Ghana’s agriculture remains rain-fed with only 3 % of its total cropping area under irrigation and <20 % of the irrigation potential is employed (Diao 2010). Ghana’s agriculture is predominantly on a smallholder basis with about 90 % of farms holding <2 ha (MOFA 2011).

Changes in climate are likely to affect Ghana’s agriculture, especially in the Upper East Region (UER), which has the highest population density, out-migration rate, and the poorest economy (Gyasi et al. 2006). Agriculture, hunting and forestry are the predominant economic activities in the region with about 80 % of the economically active population engaged in agriculture (GSS 2013). Insofar as these activities in the region are coupled with a high population density of 118.4 per km^2^, which is higher than the national density of 103.4 per km^2^ (GSS 2013), there is an increased pressure of human activities on vegetation (Ofori-Sarpong 2001).

The UER has recently witnessed periodic extreme weather shocks and weather related disasters, such as high temperatures, highly variable and erratic rainfall, long spells of droughts, late start of crops cultivation as well as livestock diseases outbreaks (Kanlisi and Arkum 2013). Such extreme weather events are expected to intensify in the region by 2080 with an average monotonic increase in temperature of 3.9 °C and an average decrease in rainfall of 18.6 % (EPA 2007; cited in: Kanlisi and Arkum 2013).

Climatic condition and economic structure make the UER an area where an understanding of farmers’ perception and adaptation practices to climate change is of crucial importance for policy decisions. For example, Ofori-Sarpong (2001), who examined the impact of climate change on agriculture and farmers’ coping strategies in the UER of Ghana using climate data at two distant meteorological stations, Navrongo (West of UER) and Bawku (East of UER), reported that the rainfall in the region was decreasing while temperature was increasing.

Nakuja et al. (2012), in examining the determinants of farmers’ adaptive capacity in dry season vegetable farming and the effects of adaptive capacities on farm income, found that adaptive capacity was influenced by the farmers’ sex and educational level, their access to land near a reservoir site, and their access to credit. Their study, which was limited to only dugouts, recommend that future research be focused on a wider view of farmers’ adaptive capacities across all types of irrigation facilities in the region. Kanlisi and Arkum (2013) from their capacity assessment of the Builsa community of UER in Ghana in light of climatic change and variability related disaster hazard experiences concluded that the community had a very low response and recovery capacity.

While these studies reveal the presence of extant challenges to climatic change throughout Ghana, they provide little in the way of solutions to the problem. Patently, none of these studies has assessed the smallholder farmers’ vulnerability and adaptation to climatic change, a population which represents a significant proportion of Ghana’s agricultural capacity.

The aforementioned literature contains several gaps that inhibit solutions to Ghana’s sustainable agricultural production when facing the presence of climatic change throughout the twenty-first century. Notably, studies to date have not focused on smallholder farmers, those representing the largest agricultural group. Also, the studies provide us with little information on farmers’ adaptation barriers, their constraints and needs. The purpose of this study is to examine farmers’ perceptions and adaptation practices to climate change and variability in accordance with actual recorded weather data of the Vea catchment in UER of northern Ghana during the time interval from 1972 to 2012. The choice of the Vea catchment is based solely on its importance to food security for much of the country. In this study, we assess farmers’ adaptation barriers and needs in overcoming anticipated shocks associated with climatic change. Despite the important role of the catchment, it is plagued with several challenges ranging from lack of rehabilitation of the aged and dilapidated canals and general infrastructure to managerial challenges which are hampering the productivity and sustainability of the Vea irrigation project. The impact of climate change could worsen the situation of the irrigation project and affect the livelihoods of the farmers and food security in the area.

## Results

Results from this study highlight temperature and precipitation trends for the catchment, farmers’ perceptions of climatic threats, their adaptations to perceived climatic threats by their farming practices (rain-fed vs. irrigation), types of farmers active in the catchment and their farming practices, their most critically perceived adaptation needs, and the types of external support accessible to farmers in order to address climatic change.

### Temperature and precipitation trends in the Vea catchment

**Fig. 1:**
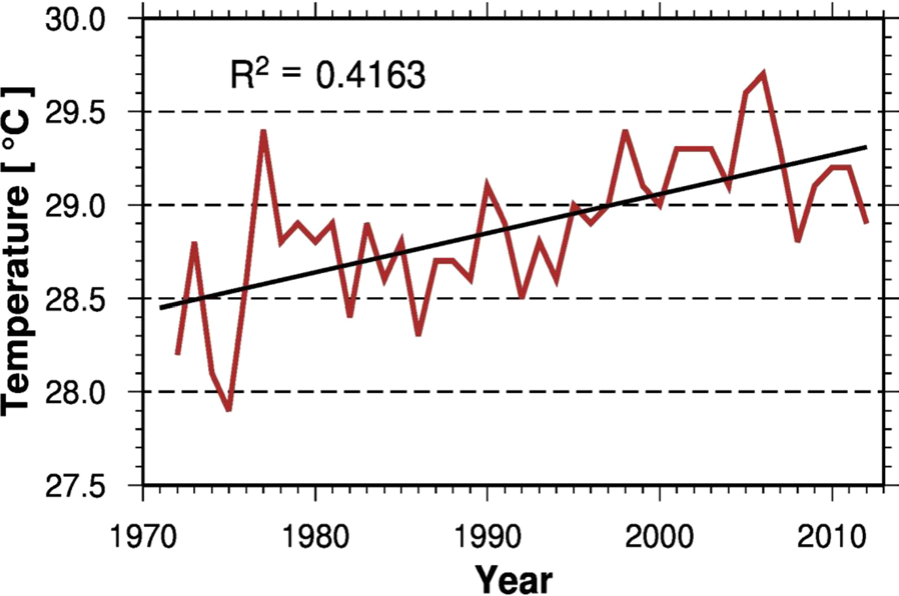
Mean annual temperature trend in the catchment (*Data source*: Ghana Meteorological Agency)

Annual temperature for the catchment over the period 1972–2012 (Fig. 1) indicates that temperature has continuously increased. The mean annual temperature over this time period was 28.9 °C. The lowest and the highest of the mean monthly temperatures within the period (1972–2012) were 25.1 and 34.4 °C and occurred in January 1983 and March 2005 respectively. The highest temperatures occurred in March and April with mean values of 39.3 and 38.5 °C respectively and the lowest were observed to occur in December and January, with long-term (1972–2012) average values of mean monthly minimum temperatures of 19.2 and 19.7 °C respectively.

A statistical analysis of temperature data for the catchment using non-parametric tests, further confirms the rising trend in temperature of the area. Both the Pettitt and Hubert Segmentation tests detected change-points in temperature time series at 1994 (Pettitt test), 1975 and 1996 (Hubert Segmentation test) at confidence level of 99 %. Mann–Kendall test likewise detected an increasing trend in temperature time series of the area at confidence level of 99 %.

**Fig. 2:**
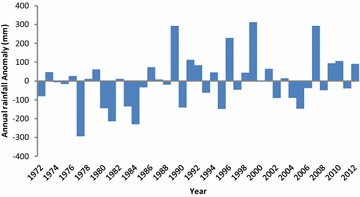
Inter-annual rainfall anomaly (deviation from long-term mean) showing variability within the study period (1972–2012) (*Data source*: Ghana Meteorological Agency)

The mean annual rainfall in the catchment for the period 1972–2012 was 957 mm per year. The lowest and highest annual rainfall amounts for the same period were 664 and 1269 mm and occurred in 1977 and 1999 respectively. Prior to 1989, rainfall in half of the recorded years fell slightly below the mean, with several droughts recorded before 1984 (Fig. 2). Post 1989, rainfall has exceeded the long-term mean excessively, with 1999 and 2007 being the wettest years on record, resulting in floods.

**Fig. 3:**
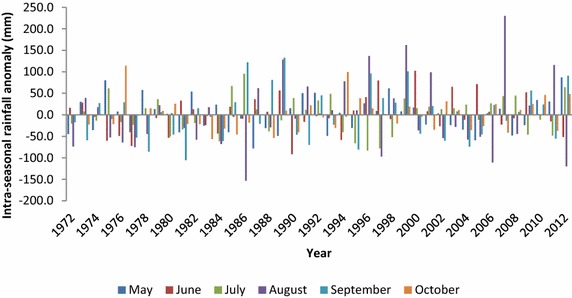
Intra-seasonal rainfall anomaly showing variability during cropping season (May–October) in the study catchment (1972–2012) (*Data source*: Ghana Meteorological Agency)

The anomalies for individual months within the growing/cropping period (May–October) (Fig. 3) indicate a general fluctuation trend in intra-seasonal rainfall distribution since 1972 with largest anomalies occurring in August and September. The maximum and minimum rainfall amounts of the peak month (August) of the wet season were 485 and 102 mm in 2007 and 1986 respectively, while the average amount was 249 mm. On average, the seasonal (May–October) rainfall totals accounted for 93 % of the total annual rainfall in the catchment over the study period.

Both the Pettitt and Hubert Segmentation tests detected no change-points in all the seven datasets (annual plus monthly rainfall for May–October) of rainfall time series at confidence level of 99 %. Similarly, the Mann–Kendall test detected no trends in all these datasets of rainfall time series of the area at confidence level of 99 %. Patently, in spite of the observed variability in precipitation, these results indicate that the average annual and average monthly rainfall levels within the growing/cropping period have not significantly changed in the catchment over the study period (1972–2012).

### Evapotranspiration

**Fig. 4:**
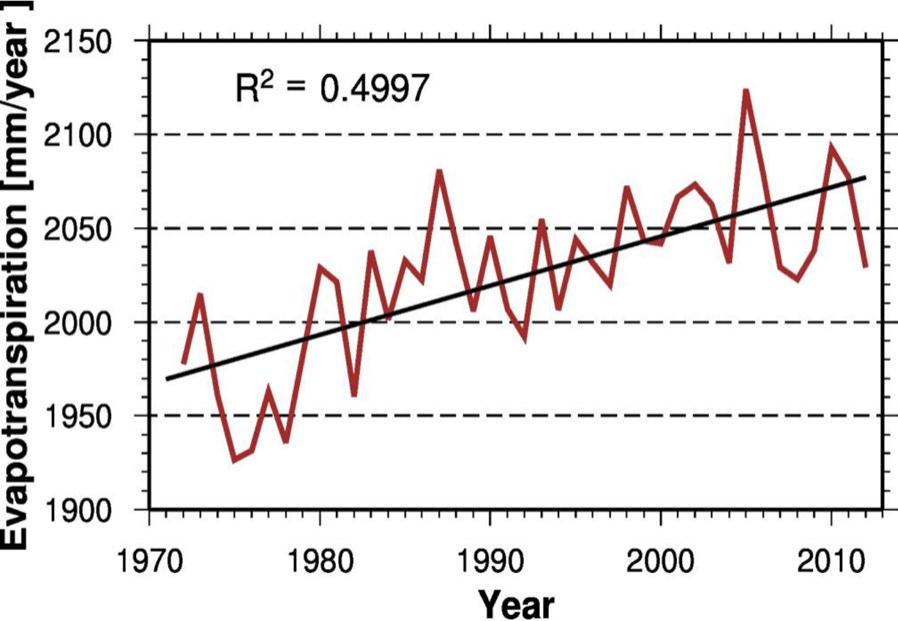
Annual evapotranspiration trend in the catchment

Conversely, given the observed increases in temperature, there is evidence of greater evapotranspiration (ET) in the catchment as illustrated by Fig. 4. The balance between precipitation and ET remains negative annually and on an increasing trend. Essentially, while precipitation, on average, remains constant, temperature and ET are increasing in the catchment.

### Farmers’ perceptions of climatic change (temperature and precipitation)

To what extent are farmers in the catchment aware of these observed changes in temperature and ET? Most farmers (89.5 %) interviewed perceived that temperature has increased over the past 30 years in the Vea catchment. However, the majority of farmers also believe (incorrectly) that rainfall decreased over the same time interval in all measures regarding amount of rainfall, duration, intensity and number of rainfall events.

**Fig. 5:**
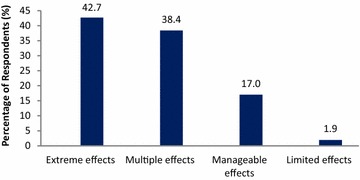
Farmers’ view of extent of negative impacts of changes in climate variables over the past 30 years

The farmers perceived various degrees of changes in climate and the effects based on their exposure levels, resilience and adaptive capacities. As is evident in Fig. 5, over 80 % of the farmers contend that the climatic effects have been extreme or notable.

The majority of farmers (97 %) perceived that they are extremely vulnerable to changes of onset of planting season, poor rainfall amount and distribution during the cropping season and also intermittent drought situations that sometimes occur during crop growth stage. According to the farmers the outlined conditions have the potential to affect the growth of the crops, their maturity and consequently could lead to reduced yields and food insecurity. Over 80 % of respondents also observed that their livelihoods face serious risk due to the increasing temperature and sporadic floods that occur in the area.

### Adaptations to perceived climatic threats by farming practices (rain-fed vs. irrigation)

Multivariate analyses conducted on farming type (exclusively rain-fed vs. some combination of rain-fed and irrigation) for their actual and/or planned practices showed that 79 % of the respondent (369 cases) practice only rain-fed while 21 % (97 cases) practice rain-fed and irrigation.

No noticeable socio-cultural differences were observed between these two groups of farmers. Almost all the inhabitants within the catchment belong to the same tribe (Frafra) with a common culture and way of doing things. The differences that exist between them appear to reflect the distances between the various communities and their location to the irrigation facility as well as the financial ability of each farmer to afford irrigation charges and land ownership. However, a few of the farmers who reside in close proximity to dug-outs within the catchment are able to practice dry season vegetable farming. Notably, the farming types differ in the frequency of use of their crop lands. While farmers using a combination of rain-fed and irrigation will use their crop lands more frequently (at least twice a year), those who practice only rain-fed will use their crop lands less frequently (once a year).

Farming practices examined across the two groups of farmers include their adjustment to temperature, adjustment to rainfall, frequency of fertilizer application, their observed opportunities, and planned capitalization on perceived opportunities. With respect to adjustment to temperature over the last 10 years, 75 % of farmers reported that they use different varieties of crop types, 17 % use a mix of crop types or mulch, and 8 % of them do something else, including selling livestock, cover cropping, and crop rotation. As an adjustment to rainfall shifts during the past 10 years, 65 % of the farmers indicated they apply fertilizer, 25 % use different types of crops, while 10 % do something else, including pesticide application, farm near rivers or hills, farm on hills, or cover cropping. Eighty-eight percent of respondents apply some form of fertilizer, 66 % of whom do so once per year. Results of the Chi square tests performed between agricultural type and practices are as presented in Table 1.

**Table 1:** Chi square tests for agricultural types (rain-fed and irrigation) by selected practices

Chi square test	*df*	Chi square	p value
Agric type with observed opportunities	3	41.94	<0.001
Agric type with planned capitalization on opportunities	2	140.25	<0.001
Agric type with adjustment to temperature	4	27.05	<0.001
Agric type with adjustment to rainfall	4	46.90	<0.001
Agric type with frequency of fertilizer application	3	46.90	<0.001

Evident from Table 1 is the finding that the two agricultural types (rain-fed vs. irrigation) do differ from one another with respect to actual or planned practices. While only 52 % of the rain-fed group would harvest flood water for irrigation, 88 % of the irrigation group would do so. Conversely, 28 % of the rain-fed group would utilize groundwater. Eighty-one percent of the rain-fed group would capitalize on increased rainfall via irrigation while 53 % of the irrigation group said that they would irrigate more than at present. While 79 % of the irrigation group would maintain or increase their use of fertilizer, only 62 % of the rain-fed group indicated their intention to do so; at 28 %, a large percentage of the rain-fed group acknowledged an intention to vary crop types without the use of fertilizer. The two groups differed markedly with respect to their use of fertilizer. Nearly 20 % of the rain-fed group either never used fertilizer or did so less than once per year. Conversely, 95 % of the irrigation group reported applying fertilizer at least once per year. A two-sample t test (t = 5.99 with 168 *df*) to assess the difference between the rain-fed (mean = 1.79) and irrigation group (mean = 2.30) means was significantly different from zero at p < 0.001.

### Farmers’ external support for adaptation

Insofar as a large percentage of the farmers depend on external support for their adaptation to the adverse effects of climatic change, no statistical differences were observed between rain-fed and irrigation agricultural farmer types regarding receipt of external support (Chi square = 0.2286, p = 0.63). Patently, 94 and 90 % of farmers relying on rain-fed and irrigation strategies respectively receive some form of support. Most of this support is delivered in the form of extension service assistance and subsidized farm inputs (i.e. the government subsidy portion of fertilizer costs). Only 3 and 8 % of the rain-fed and irrigation farmers respectively receive any type of financial or material support. In addition to the type of support received, neither the length nor frequency of support varies by agricultural type. Ninety-one percent of farmers have received support for <10 years (nearly 60 % <5 years) with 87 % of such farmers receiving support only once per year.

For a majority of the farmers who receive some form of support, the assistance is generally viewed as beneficial (Table 2). Farmers reported having benefited from the support in the following ways; increased crop and animal yields, reduced post harvest losses, improvement in household food security among others as indicated by Table 2.

**Table 2:** Farmers’ perceptions about the benefits of the external adaptation support

Benefits of the external adaptation support	Response	%
Is the external adaptation support given the respondent beneficial?	Yes	99.1
	No	0.9
Benefits of the external adaptation support to respondent	Improved yield	58.0
	Reduced hunger	19.1
	Reduced postharvest lost	16.3
	Purchase additional farm machinery	2.8
	Took another wife	1.9
	I got capital to expand my farm	1.7
	Other	0.2

Extension services and technological information are often delivered to the farmers through various channels. Such information includes weather (rainfall, temperature and wind) and technical information/assistance. Much of this support is delivered to farmers via Ministry of Food and Agriculture (MOFA) extension officers during farm visits. The analysis indicated few farmers receive such information from MOFA extension staff regularly.

Radio broadcasts have been the dominant source of information to the farmers. All local frequency modulation (FM) radio stations via the local languages provide farmers with technical information regarding farming and climate change. A few others also depended on neighbouring farmers, community leaders as well as the television broadcast (Table 3).

**Table 3:** Farmers’ sources of climatic information

Farmers sources of climatic information	Response	%
Extension officers’ regular provision of information on expected rainfall and temperature	No	65.5
	Yes	34.5
Other sources of information and technical assistance to respondent beside official extension workers	Radio	62.3
	Neighbouring farmer	23.5
	None	9.3
	Community leaders	2.0
	Relatives	2.0
	Television	0.2
	Others	0.7

### The most needed adaptation interventions

Farmers identified their most urgent needs for adaption to climatic change as irrigation development (access to water), followed by access to credit and health services (Table 4). There was apparently poor interest in the acquisition of vocational skills as most farmers did not see off-farm-jobs as an alternative to farming. All the famers interviewed (100 %) had never sought to insure their investments. The major reasons given were a lack of money or access to credit and inadequate information (Table 5).

**Table 4:** Farmers’ most needed services/investments/developments for adaptation

Perceived most needed intervention	%
Irrigation development	44.8
Credit facilities	26.9
Health services	10.9
Climatic information/extension services	8.2
Agric mechanization/subsidize farm inputs	5.4
Potable water	1.7
Electricity	1.3
Vocational/basket weaving centers (jobs)	0.4
Improved crops seeds/economic trees	0.2
Dug out (water for animals)	0.2

**Table 5:** Farmer’s reasons for not adapting some measures

Reason for not adapting some measures	Response	%
Reason for not buying insurance	Lack of money/credit facility	48.5
	Lack of information/unaware	42.7
	Don’t need it/not interested	7.5
	Not applicable here (not available)	0.5
	Don’t have trust	0.4
	Other	0.4
Reason for not finding off-farm job	Lack of information/never thought of it	27.6
	Lack of money/credit facility	23.4
	No available off-farm jobs	21.8
	Farming is better/prefer farming	12.5
	Lack of relevant skill/qualification	6.0
	I am already into off-farm job	3.6
	Planning to do so	0.9
	Because of family/marriage/socio-cultural reasons	0.7
	Other	3.5

## Discussion

The purpose of this study was to examine farmers’ perceptions and adaptation practices to climate change and variability in accordance with actual recorded weather data of the Vea catchment in UER of northern Ghana during the time interval from 1972 to 2012. During the time interval under investigation, temperature has increased in the catchment, resulting in greater ET. Yet, on average, no discernible changes in annual or monthly rainfall amounts were observed. In response to perceived changes in both increasing temperature and decreasing precipitation, farmers practice both anticipatory and reactive adaptation strategies associated with on-the-farm adaptations. The two types of farmers (exclusively rain-fed and a mix of rain-fed and irrigation strategies) were observed to differ from one another with respect to actual or planned adjustments to climatic change. While more of the irrigation group (79 %) would maintain or increase their use of fertilizer, the rain-fed group are more inclined to vary crop types instead of apply fertilizer.

These findings reinforce observations reported by other studies, particularly with respect to climate change. Ofori-Sarpong (2001), using the meteorological data from 1961 to 1998 for Navrongo, one of the meteorological stations around the study catchment, reported a steady rise in temperature. Other case studies in the West Africa sub-region specifically, and across Africa generally, report similar findings (see e.g. Zampaligré et al. 2014).The steady monotonic increase in temperature levels could have serious adverse effects on agriculture in Ghana due to a sudden drop in crop yield when temperatures exceed the optimal for biological processes (Ofori-Sarpong 2001; Bhatti and Khan 2012). Soil moisture for crops will also be affected by temperature increases, irrespective of any change in rainfall (Bhatti and Khan 2012).

Rising temperature suggests that ET is also likely to increase in the Vea catchment. Ofori-Sarpong (2001), who reported increasing ET in UER, acknowledge that such shifts could lead to a soil moisture deficit and poor crop yield, particularly when there is no corresponding increase in rainfall amount. Similarly, Obeng (2014) observed that increases in ET adversely affect crop production in the study area since grain crops (millet, sorghum, rice and maize) with shallow roots that obtain their moisture requirement from top soil remain the major crops grown in the area. The greatest impacts of climate change in the study area may stem from the associated temperature-driven ET increases and agricultural drought resulting from constant soil moisture deficits.

Farmers’ perceptions that temperature increased in the catchment corresponds with observed temperature shifts and to similar perceptions of farmers in other parts of Africa (Maddison 2007; Gbetibouo 2009; Kemausuor et al. 2011; Nyanga et al. 2011; Fosu-Mensah et al. 2012; Juana et al. 2013; Kalungu et al. 2013; Zampaligré et al. 2014). Farmers’ perception of decreased rainfall over the past 30 years is, however, inconsistent with the results from the analysis of observed rainfall data within the period as there were no trends and no break points in rainfall time series as well as there were no observed changes in variability over the study period. However, farmers’ perceptions of decreased rainfall are consistent with other reports (Gbetibouo 2009; Kemausuor et al. 2011; Zampaligré et al. 2014). Zampaligré et al. (2014) attributed the disparity between farmers’ perception of rainfall and the climatic data records to the southwards move of the isohyets observed by Wittig et al. (2007) that may have affected rainfall pattern in the Sudanian zone of which the present study area overlaps. Blench (2005), who reported a similar disparity between public opinion on rainfall changes and the historical record, attributed this difference to variation in scientific measurement of rainfall trends and drought with emphasis on meteorological drought while farmers consider agronomic droughts as explained by (Slegers 2008). According to Ovuka and Lindqvist (2000), scientists often analyse climate data at different timescales rather than those that are important for farmers and crop growth, resulting in potential variations in farmers’ perception and observed data. Additionally, the rise in ET in the catchment may have also accounted for the farmers’ perceived decreasing trend in rainfall amount.

Farmers practice both anticipatory and reactive adaptation strategies similar to what Burke and Lobell (2010) referred to as *ex ante* and *ex post* adaptive measures. These adaptive practices were linked to anticipated moisture dynamics comprising of on-farm adaptations. Farmers’ activities are partially attributable to their scepticism about off-farm alternatives, meaning they have no insurance or safety nets in case of failure (Maccini and Yang 2009). The two types of farmers (employing either exclusively rain-fed or a mix of rain-fed and irrigation strategies) do differ from one another with respect to actual or planned adjustments to climate change. For instance, while more of the irrigation group (79 %) would maintain or increase their use of fertilizer, a lesser percentage of the rain-fed group (62 %) think of doing so with more of them (rain-fed group) preferring to vary crop types instead of the use of fertilizer. Two reasons may account for this difference. First, the irrigation group may be compelled to use fertilizer due to poor soil fertility resulting from the frequent use of the land. Second, the irrigation group may have a relatively stronger financial standing than the rain-fed group by virtue of their additional income generated from the dry season irrigation farming. While no statistical differences were found between rain-fed and irrigation agricultural types regarding receipt of external climate change adaptation support, the external adaptation support received by farmers varied in terms of frequency of access and content of support such as extension services, material inputs or financial support. Yet other studies have shown that access to credit is influential in adapting to climatic change (Butt et al. 2006; Antwi-Agyei et al. 2012; Fosu-Mensah et al. 2012; Nakuja et al. 2012).

Most of the technical assistance necessary for effective farmer adaptation arrives either too late or in some cases not at all. This outcome has resulted in many farmers lacking reliable climate adaptation information, particularly about the onset and cessation of rainfall (Antwi-Agyei et al. 2012). A majority of the farmers believe that access to an irrigation facility will serve their major need. This is consistent with findings by Antwi-Agyei et al. (2012) that indicated that irrigation facilities were crucial for climate-sensitive (rain-fed) agriculture dependent farmers in the area, as means of reducing their vulnerability to climate change. Some farmers believe that access to credit is all that is needed to avert potential negative impacts of climate change. Nakuja et al. (2012) reported that access to credit is the primary determinant of farmers’ adaptive capacity in parts of the UER.

### Relevance of the present study to the broader community

Evident from the findings of this study, as well as others, is the contention that climate change will affect agricultural systems ranging from commercial to subsistence/smallholder farmers in all countries (FAO 2008). The scientific evidence and the experiences of nations and communities have shown that adaptation measures are increasingly necessary in order to alleviate the adverse effects of projected climate change (Kirsty 2009; UNFCCC 2011). For instance, in countries where agriculture serves as the main pillar of the national economy, such as Ghana and other developing countries, adaptation in smallholder farming systems becomes crucial for food security and poverty reduction, as well as for other socio-economic and structural development (FAO 2010).

The exchange and sharing of knowledge, information and experiences of different adaptation practices at all levels of the global community, including the regional, national and local community levels, have therefore become necessary for the timely implementation of effective adaptation actions (FAO 2011). This focus was the core objective of the Nairobi work programme on impacts, vulnerability and adaptation to climate change adopted at the eleventh session of the Conference of the Parties to the United Nations Framework Convention on Climate Change (UNFCCC 2011). The motivation for such international adaptation initiatives is the conjecture that the knowledge, information, experiences and lessons learned about adaptation from one community or region could be useful for others (Kirsty 2009; UNFCCC 2011; Chishakwe et al. 2012). The availability of a wide-range of observations and assessments of local community level adaption practices and lessons learned about adaptation from different regions, is essential for effective adaptation planning, particularly at the local community levels where capacity to respond to climatic threats depends in part on this information (Kirsty 2009). Such knowledge and information sharing are particularly important for regions and communities with similar climate patterns as well as similar socio-economic and socio-cultural values in the developing and least developed countries. Such information, experiences and lessons are also important for global, regional and national initiatives and networks in promoting, streamlining, and prioritizing the best adaptation practices while preventing maladaptive practices in the development of policies and strategies for the wider regional and national community uses.

Findings from this study are arguably of interest to parties and partner organizations of a global learning platform for the exchange and sharing of knowledge and experiences on the four key components of adaptation to climate (UNFCCC 2011). These four components of adaptation include (1) the assessment of climate impacts and vulnerability, (2) planning for adaptation, (3) the implementation of adaptation measures, and (4) the monitoring and evaluation of adaptation actions. Findings from this study might therefore potentially contribute to the global experiences and lessons of the first two of UNFCCC adaptation objectives (components). Results from this study may also improve our collective understanding of smallholder farmers’ perceptions of climate change and their local and community-based adaptive strategies, barriers, constraints and needed supports, as well as serve as a contribution of knowledge to help prevent maladaptive practices that act as counterproductive to environmental protection.

A broader international platform for information, experiences and lessons learned from climate change adaptation mechanisms include the local coping strategies database platform of UNFCCC that serves as reference material on adaptation practices of long-standing coping strategies from local communities across the globe as identified from the various case studies (UNFCCC 2011). Within the West Africa sub-region, limited studies (e.g. Zampaligré et al. 2014) have compared farmers’ perceptions on climate change with analysed trends of climatic variables (temperature and rainfall) and adaptation practices similar to the current study. Lacking in this regard are investigations of smallholder farmers’ adaptation barriers to building resilience to climate change. The findings of our study address this issue, providing potential value to other agricultural areas of the West Africa sub-region along the Sudan Savanna and Guinea Savanna Ecological Zones with similar climate patterns as the study area, especially across the northern parts of Togo, Benin and Nigeria, as well as the southern part of Burkina Faso. Besides similarities in the agricultural practices and the major crop types used (cereals, e.g. millet and maize), the socio-economic, socio-cultural and ecological values of the inhabiting communities along these two Ecological Zones are closely related, potentially reinforcing perceptions about climate change, adaptation options and future expectations or needs of the mostly farming populations across these areas. The results of this study may, therefore, serve as larger set of lessons learned with respect to stakeholders and policy-makers in these two ecological zones of the West Africa sub-region and other parts of Africa with similar rainfall and temperature patterns as the study area.

### National agricultural sector policy in Ghana: some recommendations

Ghana’s agricultural policies are contained in the Food and Agriculture Sector Development Policy (FASDEP), as a continuation of what the Accelerated Agricultural Growth and Development Strategy (AAGDS) initiated as a growth engine for the private sector (MOFA 2007). The current policy document for the Agricultural sector is FASDEP II (MOFA 2007). The FASDEP II became necessary after poverty and social impact analysis (PSIA) showed that the FASDEP I, which had been developed in 2002, was not capable of accomplishing the desired impact on poverty (MOFA 2007). As a result FASDEP II was developed to provide a level environmental ground for all categories of farmers with a special focus on the poor and vulnerable groups. The FASDEP II adopted wide range policies to address challenges within the agricultural sector in Ghana, including human resource and managerial skills; natural resource management; technology development and dissemination; infrastructure; market access; food insecurity and irrigation development and management (MOFA 2007).

Six main policy strategies were identified to address challenges in the agricultural sector but for the purpose of this research, only policies related to this study are reviewed. Among the activities under the food security and emergency preparedness strategy are development of irrigation schemes, development of high yielding and short-duration crop varieties and promoting proper methods of managing harvest. Through this strategy the nation seeks to enhance early warning systems and preparedness for disaster to avoid loss of harvest. Another key strategy is to increase farmers’ incomes. Activities such as diversification of crops, vegetables, small ruminants and poultry are also considered in the strategy.

The policy apart from plans to enact and enforce appropriate practices is intended to facilitate the development of agriculture in Ghana. Toward this end, the policy seeks to promote the development of community level land use plans and enforce their use. Earmarked interventions include research into development and industrial use of indigenous crops and livestock by producing certified seeds and breeds for farmers to adapt. Extension service development and delivery is to be improved to identify appropriate methods of delivering equitable services to farmers.

As noted above this policy also seeks to create a level playing environment for all farmers and one of the ways of doing this is to expand irrigation facilities and increase the production capacity of the current schemes. Consequently, there is now the realization that achievement of national agricultural sector policy objectives is closely linked to the national water resources sector policy which, historically, has always been informed by international development objectives such as millennium development goals (MDGs), New Partnership for Africa’s Development (NEPAD) and others. Among several special focus areas of Ghana’s national water resources policy objectives are water for food security, capacity building and climate variability/change. To make water available for food security the water sector policy seeks to support micro-irrigation and valley bottom irrigation in communities, enhance the capacity of district assemblies (DAs) to support how communities operate and maintain irrigation facilities and promote the efficient use of fertilizers to reduce pollution of water bodies and ensure water conservation (MWRWH 2007). To that effect, Buffer Zone Policy has also been designed to streamline all the existing ineffective and fragmented regulations on buffers bordering water bodies in the country and to provide comprehensive strategies that would ensure sustainable creation of vegetative buffers for the preservation of all water bodies and river systems (MWRWH 2011).

Agricultural mechanization and financing are major issues also captured in the agricultural sector policy. Here initiatives such as developing appropriate machinery and equipments and promoting access to them would be all implemented. With very high reliance on rainfall and increase unpredictability of rain, it is obvious that adequate and efficient irrigation facilities are required to facilitate agriculture mechanization. Small irrigation dams, dugouts and renovation of non-functional dams and canals should be undertaken. This should be accompanied by capacity development of smallholder farmers and farmer based organizations and training in climate-smart agriculture practices. On financing, access to credit by all categories of farmers is to be ensured at all levels through the implementation of several activities. Facilitating access to credit should be channeled through financial institutions who have gained considerable expertise over management of credit to farmer-based organizations. This should be done at lower interest rates, and be cashless in nature to reduce farmer’s diversion of farm inputs (i.e. spending it on irrelevant things) and enhance commercialization of smallholder farmers.

The successful implementation of these policies however requires the commitment of government and its agencies in terms of action, effective resources allocation, coordination and nurturing the necessary linkages between stakeholders particularly to the research sector for the requisite information and data required for review and implementation of policies. The response and inputs from the research sector to the policy-makers is critical for the realization of the policy objectives particularly at the farmer level. An important condition to be met to enable farmers to benefit and take advantage of the policy interventions is mainstreaming these policy objectives into the plans of the DAs where the need for survey results as these increasingly arise. National policies of each of the sectors therefore, seem to have made adequate provisions for the inclusion and implementation of research recommendations. These initiatives integrated into the District Medium Term Development Plans should be accorded necessary budgetary allocations to fund the findings and initiatives that promote and encourage the use of economic indigenous and climate-tolerant seed varieties necessary to respond to the impacts of climate change. The results of this survey are that such inputs from research sector required to provide direction for the successful implementation of the agricultural sector policy and therefore could be supportive to the achievement of the policy objectives.

## Conclusion and policy recommendations

The Vea catchment is certain to face a long-term climactic shock as temperature and ET increase throughout the region. Results of our study show that farmers are generally aware of climate change, although in very subjective terms as their understanding of it is directly linked to the outcome of their daily livelihood activities. Even though most of the farmers could not explain what climate change is, they could give a description of it based on their assessment of increases in temperature and changes in the duration, intensity, and timing of rainfall. Their observations about changes in climate were generally consistent with analyzed observed temperature data, but partly differ from results of analyzed observed rainfall. Farmers’ perceptions of decreasing rainfall could be influenced by the rising temperature-driven ET. Nonetheless, perception about frequent droughts and sporadic floods could be attested to by the observed data. In spite of farmers’ knowledge of climate change, its effects and alternative livelihood chances, they are challenged in pursuing effective coping strategies because of poverty, inaccessibility to loan facilities, lack of irrigation facilities, et cetera.

There is a major shortfall of extension services/information and technology dissemination as well as farmers’ access to organisations such as farm input suppliers, food purchasers, et cetera. With limited governmental interventions, the farmers appear to lack sufficient capacity to avert the environmental threat. Government policies should, as a matter of priority, ensure that smallholder farmers have access to flexible and affordable credit/loan facilities in order to embark on effective coping strategies in response to the changing climate. In view of the important role the extension services play in ameliorating the adaptive capacities of farmers to climate variability/change, there is a need for the formation of a modern agricultural extension system of four actors (public agencies, private service providers, farmer groups and non-governmental organizations) and ensuring smooth coordination among these actors. This system will provide farmers with a platform to participate in decision making based on an improved knowledge base and stronger partnerships with private actors who might facilitate their access to a range of services such as farm inputs, loan facilities, product marketing, etc.

Insofar as irrigated agriculture plays a pivotal role in building the adaptive capacities of famers against the adverse effects of climate variability/change and was identified by most farmers as their most needed adaptation intervention, provision of adequate and efficient irrigation facilities should be embarked on as a principal measure for building farmers’ resilience to these effects. This should be accompanied by organised training and demonstrations for farmers on efficient use of such irrigation facilities that include efficient and productive use of water.

The use of different variety and crop types has already been employed by majority of farmers as an adaptation option to climate variability and change, which is an indication that farmers see this option as an effective measure to ameliorate their adaptive capacity. Farmers should therefore be assisted with provision of drought-resistance crop varieties. A broader dissemination of forecasted climate conditions through annual community-based sensitisation is also of great need in the study area.

## Methods

### Overview of the study area

**Fig. 6:**
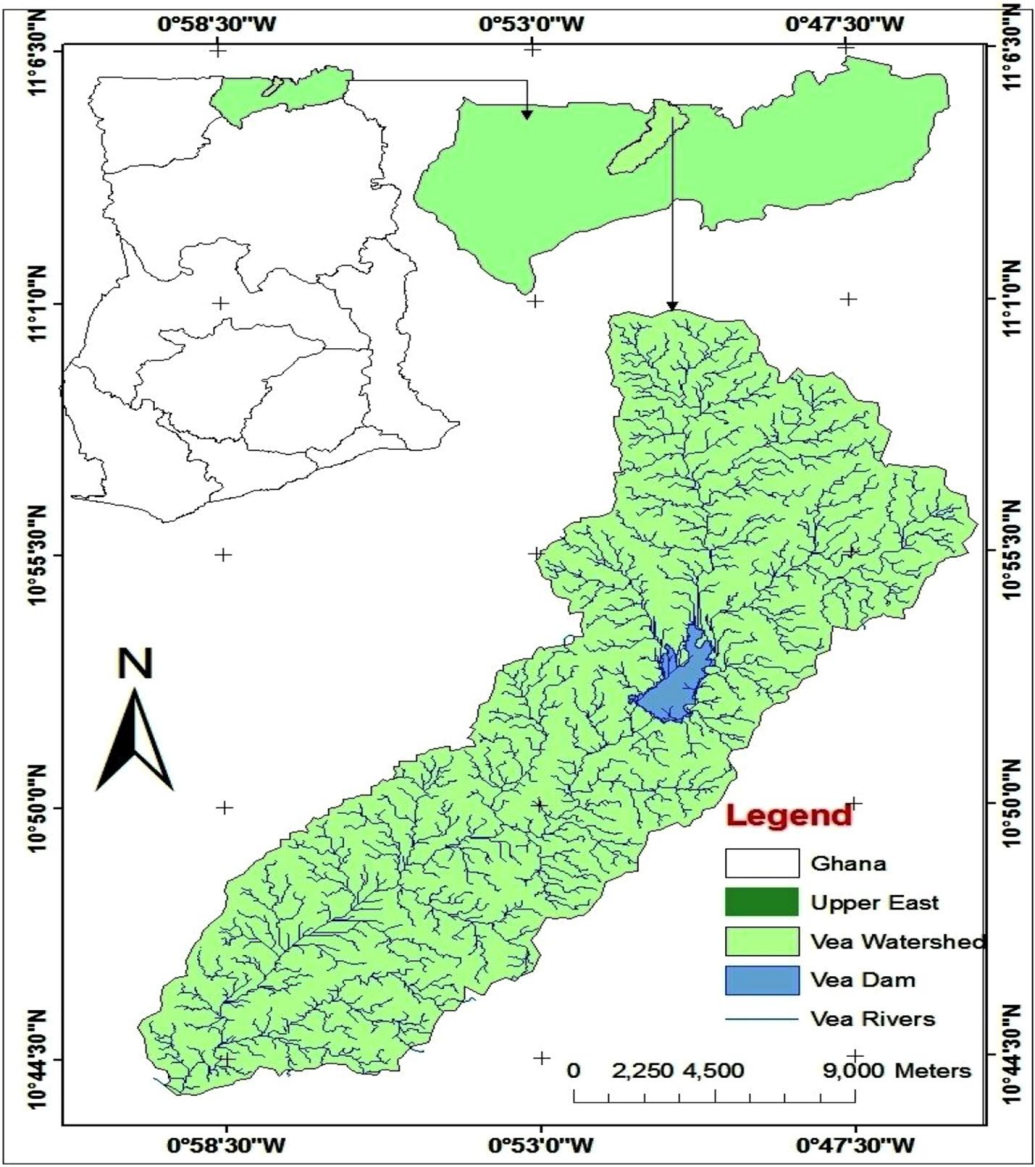
Map of the Vea Catchment (305 km^2^) showing the Vea dam irrigation reservoir

Research for this study was conducted in the Vea catchment which is part of the White Volta Sub basin and located in the UER of Ghana extending to the border between Ghana and Burkina Faso, covering an area of 305 km^2^ (Fig. 6). The catchment area is located between longitudes 0°45′0″–1°0′0″W and the latitudes 10°42′30″–11°02′30″N extending over Bongo and Bolgatanga districts with a smaller portion over the south-central part of Burkina Faso. It falls within a semi-arid agro-climatic domain and across three agro-ecological zones: Savanna and Guinea Savanna zones in Ghana and the North Sudanian zone in Burkina Faso (Ibrahim et al. 2011). The area is marked by only one rainfall/growing season from May to October and peaks in August characterized by erratic rainfall and often associated with floods and droughts, followed by a long dry season that often spans from November to April (Gyasi et al. 2006). The average temperature within the catchment ranges between 28 and 29 °C with the average annual rainfall and annual average ET about 950 mm and between 2000 and 2050 mm respectively (Ibrahim et al. 2011).

### Data

#### Acquired meteorological data

Historical long-term daily observed rainfall and temperature data for four meteorological stations, in and around the study catchment from 1972 to 2012 (41 years), were obtained from Ghana Meteorological Agency.

#### Survey data

The survey data was obtained using a designed household questionnaire (see “Appendix”).

### Variables

Key climatic variables analyzed in this study include annual temperature, annual rainfall, monthly rainfall and ET.

The catchment area rainfall and temperature were computed using data from the four meteorological stations. There are several methods available for determining average rainfall over any area of interest, but selecting any of these methods depends on its suitability for a particular study or the distribution of the rain gauge stations in the study area (Ward and Elliot 1995). The Thiessen Polygons method is commonly preferred for study areas where rain gauges are not evenly distributed (Ward and Elliot 1995) as in the current study. The daily areal rainfall and temperature used in computing the monthly and annual values required were therefore computed using the obtained daily data from these stations for the study catchment area by Thiessen Polygons interpolation method and Simple Arithmetic average method respectively. ET was subsequently determined as described in the following subsection using the temperature data obtained.

The daily areal rainfall for the study catchment was determined as a weighted average of the observed daily rainfalls of the four selected stations in the following relation (Eq. 1) as described by Ward and Elliot (1995):

1 $${\text{P}} = \frac{{\mathop \sum \nolimits_{i = 1}^{n} A_{i} \times p_{i} }}{{\mathop \sum \nolimits_{i = 1}^{n} A_{i} }}$$

where P represents the average depth of rainfall in the catchment of total area of $\sum\nolimits_{i = 1}^{n} {A_{i} ,}$ and *A*_*i*_ being the area of the *i*th polygon with rainfall depth of *p*_*i*_ in that polygon.

The survey questionnaire variables used in the analysis were under the following general categories: agricultural type, agricultural practices, climatic adaptation strategies, and support.

### Estimation of evapotranspiration

The trend of potential evapotranspiration (PET) over the study period was examined. Thornthwaite’s (1948) method of estimating PET was employed as follows (Eqs. 2–4):

2 $${\text{PET}}_{t} = 16\left( {\frac{10T}{I}} \right)^{a}$$

where PET_*t*_ is the monthly PET (mm); T is the monthly mean air temperature (°C); *a* = $(6.75 \times 10^{ - 7} )I^{3}$ − $(7.71 \times 10^{ - 5} )I^{2}$ + $(1.79 \times 10^{ - 2} )I$ + 0.49239; and *I* is the annual heat index computed from monthly indices as follows:

3 $$I = \mathop \sum \limits_{i = 1}^{12} \left( {\frac{{T_{i} }}{5}} \right)^{1.514}$$

T_i_ is the mean air temperature (°C) for month *i*; $i = 1, \ldots ,12.$

Remarkably, PET_*t*_ is the theoretical evapotranspiration based on 30 days and 12 h of sunshine per day. Thus actual ET (ET_*a*_) for a particular month with mean temperature *T* °C was obtained by:

4 $${\text{ET}}_{a} = {\text{PET}}_{t} \left( {\frac{{DL_{d} }}{360}} \right)$$

where D is the number of days in the month and *L*_*d*_ is the average number of hours between sunrise and sunset in the month. Finally, annual actual ET for each year was obtained by summing up ET_*a*_ for all the 12 months in the year.

### Research design and sampling

An intensive reconnaissance field survey of the study area was administered between June and July, 2013. This effort sought to obtain first-hand information on geographic, hydro-graphic and physical characteristics of the area and most importantly the various farming systems/methods and any adaptation measures practiced. The reconnaissance field survey was also intended to obtain information about the farm locations, crop types, land use and land cover types prior to a detailed survey.

Based on the field survey, a structured household questionnaire (see “Appendix”) was designed into five main sections and covered information on demography, farming systems, farmers’ perceptions on climate change, concerns for changes in climate, their adaptation practices and external support for adaptation, barriers to adaptation, future adaptation plans as well as the support they needed to build resilience. Sections of the questionnaire designed for the current study were adopted from a farm household’s questionnaire by Gbetibouo (2009). The questionnaire was administered to farmers with the help of 14 Agricultural Extension Agents (AEAs) of the MOFA in the Bolgatanga municipal and Bongo districts.

The study area is divided into agricultural operational areas supervised by an AEA. The operational areas consist of enumeration areas (EAs), i.e. (special areas where intensive data collection is carried out with the farmers for annual agricultural performance assessment). Six out of eleven such EAs were chosen for this study in a stratified manner that maximized representation and generalizability to farmers in the area. The selected EAs include two each from upstream, middle and downstream of the catchment. Farmers within each EA are registered (listed) by AEA and constitute a farmers’ group who share ideas and resources.

Selection of farmers was conducted via a purposive sampling method in which adult farmers of age group of 50+ years with at least 30 years farming experience were selected in each EA using MOFA’s holders list. This method of selecting a sample frame was preferably employed based on the idea that it is one of the ways of getting the best information through selecting people most likely to have the experience to provide quality information on the research topic (Denscombe 2010). The age group of 50+ was targeted because the focus of this study interval spans over the last 30-year period as required for meteorological data time series analyses.

The farm holders list (inclusive of both sexes) from the MOFA in the Bolgatanga and Bongo districts showed that the total number of registered farmers in all the 11 EAs of the catchment is 2921. A total number of 1102 of these farmers are aged 50+. From this population of farmers aged 50+ in each EA, the sample size for each of the selected EAs was determined using an online sample size calculator as recommended by Denscombe (2010). With the online sample size calculator by Creative Research Systems (2012), which applies the formulae (Eqs. 5, 6) by Jerrold (1984), the sample sizes of the entire listed farmer population (2921) and farmers aged 50+ (1102) were found to be 340 and 285 respectively at confidence level of 95 %. However, the sum of the sample sizes of farmers aged 50+ in all the selected EAs was 466, which is well above the sample size (340) of the entire listed farmer population of the catchment. To maximize generalizability and reduce potential challenges with non-response, 466 was selected as the target number. Determining sample size separately at each of the selected EA was done to ensure uniformity and representativeness of the responses at all the selected EAs and the catchment at large.

5 $${\text{ss}} = \left( {Z^{2} \times (p) \times (1 - p)} \right)/c^{2}$$

where ss = sample size, Z = Z value (e.g. 1.96 for 95 % confidence level), p = percentage picking a choice, expressed as decimal, c = confidence interval, expressed as decimal.

Correction for finite population gives:

6 $${\text{new}}\,{\text{ss}} = ss/[1 + (ss - 1/pop)]$$

where pop = population.

The structured questionnaire (see “Appendix”) was administered by random sampling technique using random number generator software employed by MOFA for similar surveys. The random generator was applied to each of the six selected EAs for selection of the respective number of respondents in each EA. Farmers that make up the sample size in each EA were randomly selected based on their serial numbers in the list and then the names and houses of the randomly selected farmers to be interviewed in each EA were identified by these randomly selected serial numbers. This strategy enabled the AEAs who already know all the communities and the houses of these farmers in each EA to easily reach them for interview.

A field survey approach of face-to-face interview method of questionnaire administration was adopted to maximize the response rate (Denscombe 2010). A total of 466 household questionnaires were administered to farmers in the study area. To obtain reliable responses on climatic information, the questionnaire administration was conducted using appropriate local terms in the common local language (Frafra) spoken by the people in the study area. From a household of more than one farmer, only one farmer was interviewed to avoid bias as observed by Kemausuor et al. (2011). The farmers in the area include mainly those who practiced rain-fed only and those who practiced both rain-fed and irrigated agriculture. Interviews of farmers were conducted between August and September, 2013. The data gathered were processed and statistically analyzed using the Statistical Package for the Social Scientists (SPSS 16.0) software and the results interpreted.

### Socio-cultural structure and the representativeness of the sample frame

The study area is composed entirely of rural and subsistence farming communities with similar social and cultural characteristics as well as common socio-economic interests. Just like other farming communities in Ghana and elsewhere in West Africa, members of the society are under strong socio-cultural influence to behave and do things in relation to the culture of the society. This includes the types of agricultural practices and their adjustments to changes in climatic variables. In these farming communities, most activities and practices including agricultural practices are carried out in unison and under strong influence related to the culture of the area. As observed by FAO (1985), in farming communities like those of the study area, an unmarried man works on his father’s farm until he marries before he is expected to start farming on his own field and if it is customary for farmers to cultivate by certain methods, people grow up believing that those are the only correct methods of cultivation. These customary practices and influence are passed on from the older people in the communities.

The inhabitants in the study area are indigenous people and other farmers who are younger (i.e. below age 50 years) are the children of the older farmers. These young farmers started farming with and under the direction and supervision of their parents when they became of age to assist their parents on the farms. In most of these rural farming communities in the area the youth are made to assist in the farm work by their parents at about age of 15 years and continue until they marry and form households of their own.

The communities in the study area are characterised by compound houses with often several households in a compound (often extended family of the older farmer and the sons’ households) and who share ideas, resources and do things in common including respect for ideas and decisions of the older people. The entire society of the study area is customarily like many others observed by FAO (1985) to be where elderly people are greatly respected and their advice is carefully listened to and taken very seriously. The potential influence of older farmers’ strategies on all farmers’ strategies is, therefore, further enhanced by the fact that the older farmer is the overall head of all household heads in the compound and his/her ideas/decisions are respected by all in the compound. Older farmers, by virtue of this social organization, tend to have much more influence over other farmers in all respects, particularly in agricultural practices with regards to perceptions and adaptation strategies.

The sample frame was, among other factors, chosen based on the understanding of the social and cultural background of the farmers in the study area through the reconnaissance study.

Nonetheless, some younger farmers, by virtue of their exuberant nature, could possibly conceive some form of ad-hoc adaptation strategies differently from the older farmers as was observed during reconnaissance survey. While the older farmers practice accepted on-farm adaptation strategies seriously because that is the only way they can sustain their livelihood, some of the young farmers think differently. Some young farmers in communities where dry season farming opportunities are absent prefer to go to big towns and cities in the southern Ghana, especially in the dry season, to engage in temporary labour for cash. The older farmers who do not have access to dry season farming intensify their animal production activities.

### Selection and training of interviewers

The agriculture extension agents (AEAs) were selected in the administration of the survey based on their familiarity with farmers in the communities. During the reconnaissance field survey it was unearthed that majority of the farmers were not willing to freely disclose information to us simply because we were strangers. On the other hand, they freely divulged information to the AEAs who accompanied us the second time on reconnaissance field survey. The AEAs are the Government personnel who work directly with the farmers to deliver extension services and technologies as well as send identified research issues from the farmers for the attention of research institutions. The AEAs are tasked to work with farmers in designated areas called operational areas under the supervision of superior officers. The AEAs who have EAs in their operational areas, are tasked to do this extra work of data collection. These AEAs were given special training in data collection techniques and minor analysis by the MOFA. Therefore, all AEAs who do this special work have been trained and they have been collecting quality data for MOFA and other institutions.

All the AEAs who collected data for the current study have been working in the EAs for three or more years and were involved in data collection for previous studies in the area conducted by students of West African Science Service Center on Climate Change and Adapted Land Use (WASCAL) and other research institutions. These AEAs can, therefore, be said to have experience in data collection including data collection for scientific research purposes.

The AEAs employed for the conduct of the interviews and data collection in this study were adequately briefed on the purpose of the study and vigorously trained and tested on their role for 2 days before departing them to the field. The aim was to reduce any inconsistency or variation and possible biases in the data collection process to the barest minimum. On the first day of training, each AEA was carefully taken through the questionnaire (see “Appendix”) and expected responses/information, trained on how to behave in neutral ways to avoid their influence on the answers, how to assure respondents of the confidentiality of their responses, how to present the questions for accurate answers, followed by pre-testing (field test) of the questionnaire with a sample of potential respondents in the neighbouring catchment (Atankwidi). After the evaluation of the training and the results of test data collected, the questionnaire was reviewed and revised, then the entire training exercise was repeated the second day and final evaluation done before their departure to the field on the third day. They were regularly monitored (visited) on the field each day until the data collection was completed.

### Minimizing and assessing possible biases of interviewers

Despite our efforts at standardization via trainings and characteristics of the AEAs, biases in data collected cannot be completely ruled out due to human errors or weakness from interviewer and/or respondents. That is the normal challenge as with almost all survey studies and therefore provision is usually made for margin of error. We addressed the extent to which interviewer bias may have influenced the results in two distinct ways. First, as discussed previously, interviewers were selected based on their experience and trained to minimize bias through a formal process as explained in the preceding subsection. Second, we conducted an exploratory analysis to determine if interviewers differed from one another on participants’ responses to key survey questions. Insofar as the interviewers were drawn from different agricultural operational areas, with each operational area representing a different agricultural type profile, we can assume, based on our findings that farmers who emphasize rain-fed approaches will differ from farmers who utilize irrigation techniques. To assess the likelihood that interviewers may have biased the results, we examine the extent to which interviewers have notably different responses for the same type or combination of farmers. An ANOVA and Tukey’s test were conducted for the two groups of farmers (rain-fed only and a mix of both rain-fed and irrigation) with respect to the various interviewers and key response variables to examine the possible differences in the responses between interviewers. An examination of Chi square tests for the agricultural type and practice variables was additionally conducted.

**Fig. 7:**
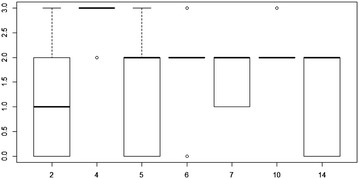
*Box plot* of response variation in reported fertilizer use for interviewers of farmers employing only rain-fed techniques

Seven of the fourteen interviewers only interacted with rain-fed farmers while seven include a mix of both rain-fed and irrigation farmers. These two groups of farmers were examined separately on the assumption that interviewers who interacted with the same type of farmers should, on average, have comparable responses. An ANOVA conducted for each of the two groups between interviewer and fertilizer use produces an F-ratio observed to be significantly different from zero. Essentially, the average response of one or more interviewers differs significantly from the others. A Tukey’s test of group differences reveals that, with respect to the rain-fed group, interviewer #2 differs from interviewers 4, 6, and 10 while interviewer #4 differs from interviewers 2, 5, 6, 7, 10, 14. With respect to the seven interviewers containing a mix of rain-fed and irrigation farmers, the mean response of interviewer #1 differs from that of interviewers 3, 8, and 12. Box plots of these tests are presented in Fig. 7 (rain-fed only) and 8 (mix of rain-fed and irrigation).

An examination of Chi square tests for the Agricultural Type and Practice variables revealed some additional differences. Notably, among the interviewers of the rain-fed group, while no differences were observed by agricultural type, interviewers 10 and 14 had a heavier use of responses in the “other” category. Among interviewers of the mixed group of farmers, interviewer 12 made much more use of responses in the “other” category. These differences could arguably reflect idiosyncrasies associated with a particular locale in which the interviews were conducted. For example, fertilizer use might be more or less pronounced among farmers in a particular locality depending on the type of crop most used there, e.g. maize which require more fertilizer due to poor soil.

**Fig. 8:**
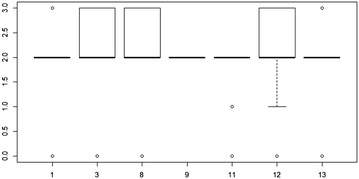
*Box plot* of response variation in reported fertilizer use for interviewers of farmers employing a mixture of rain-fed and irrigation techniques

An analysis of support by interviewed farmers identified some minor differences, which were statistically significant at p < 0.05. Namely, of the interviewers who interacted only with rain-fed farmers, two (interviewers #10 and #14) reported five farmers who did not receive support while the other five interviewers identified either one or none of their respondents who reported an absence of support. With respect to the interviewers who interacted with a mix of rain-fed and irrigation farmers, two (interviewers #8 and #14) reported 14 and 9 farmers respectively without aid while the remaining five interviewers reported two or fewer farmers without any acknowledged aid. In total, only 40 of the 466 farmers interviewed reported that they did not receive support of any kind. The similarities in these observations suggest that interviewers, on average, were quite consistent with the types of responses they received, an indication that either all of the interviewers or none of the interviewers introduced bias into the farmers’ responses.

So is there evidence of interview bias? In total, the direct answer would appear to be no. The various external supports given to farmers are independent of the type of farmers and farmers’ choices but somewhat dependent on the specific goals and the interests of each organization that provides support. The main government support provided through extension services and subsidies on farm inputs such as fertilizer, as well as all other government external supports target all communities except where a particular support is deemed unsuitable based on geophysical characteristics of the recipient community with respect to crop type. Conversely, external supports provided by organizations other than the government do not typically target all communities. Instead, such supports are more crop-specific and sometimes dependent on geophysical characteristics of the recipient community. Such organizations only receive technical advice from MOFA extension officers. Insofar as there is typically a lack of coordination among these organizations in rendering these adaptation supports in the area, most farmers are usually not consulted or involved for their opinion in the initiation of such supports. Nonetheless farmers tend to view any type of support as an opportunity. The spatial and temporal variations of such supports are therefore independent of the farmers’ choices or opinions, but solely dependent on the goals, interests and decisions of the organizations providing such supports. The spatio-temporal variation in the kinds of external support observed is therefore likely independent of the types of farmers in the study area and/or the interviewers responsible for the collection of data.

### Analyses

#### Trend test and change-point detection for the meteorological data

Pettitt test (Pettit 1979) for comparing two segmentations of data time series and widely employed for detecting changes in hydro-meteorological time series data (Mu et al. 2007; Ma et al. 2008) was used in this study. This was followed by examining the long-term trends in climatic time series data of the catchment using Mann–Kendall test statistics (Mann 1945; Kendall 1975) which is more robust when dealing with skewed data and outliers in a data series (Onoz and Bayazit 2003). Mann–Kendall analysis method was particularly used to detect linear trends in temperature and rainfall time series. Hubert Segmentation test (Hubert and Carbonnel 1987) was also used for multiple change-points detection for both temperature and rainfall time series of the study area. The software package, KhronoStat, developed at the Science House of water of Montpellier (Lubes et al. 1998), within which the three tests (Pettitt test, Mann–Kendall test and Hubert Segmentation test) are embedded, was used for the current study.

Our interest was in analysing annual mean temperature time series and seven aspects of the rainfall time series. For that reason, seven different datasets were derived from the primary daily dataset for analysis of annual and seasonal aspects of the rainfall time series. These include the time series of annual total rainfall amount and time series of monthly rainfall amount of each of the 6 months in the growing season of the catchment (May–October).

#### Rainfall variability analysis by sample variance: F-ratio and F-distribution method

A test for an increasing (or decreasing) variance trend was carried out to assess changes in variability of rainfall time series over the study period. Seven different datasets of rainfall time series of the study period obtained as described in the preceding sub-section were each divided into two arbitrary parts (e.g. 1972–1992 and 1993–2012) and changes in variances between the two samples examined using F-ratio (ratio of variances) and F distribution test (comparing the variances of two independent samples by testing whether or not the two sample variances are equal) as described by Foltz (2013). The question was, is the variance ($S_{x}^{2}$) of the first data sample (1972–1992), the same as that ($S_{y}^{2}$) of the second sample (1993–2012) or different? That is: $S_{x}^{2} = S_{y}^{2}$?

We first compared the relative size of the two variances using an F-ratio with the largest of the two sample variances as numerator and the smaller of the two sample variances as denominator.

${\text{F}} = \frac{{S_{x}^{2} }}{{S_{Y}^{2} }};$ the F-ratio, where $S_{x}^{2}$ = larger sample variance and $S_{y}^{2}$ = smaller sample variance.

This was then followed by testing to find out if statistically, significant difference exists between the two sample variances using F-distribution test. Notably, when independent random samples, *n*_*X*_ and *n*_*y*_ are taken from two normal populations with equal variances, the sampling distribution of the ratio of those sample variances follows the F-distribution.

Each of the numerator and denominator of the F-ratio has degrees of freedom given as, *n* − 1, where *n* represents the sample size of each of the two samples. Thus the degrees of freedom of both the numerator and denominator are *n*_*X*_ − 1 and *n*_*y*_ − 1 respectively. The critical F-value on the F-distribution which marks the boundary between the lower 95 % (non rejection region) and the upper 5 % (rejection region) is determined using the F-table (or digitally) with chosen significance level (*α*), numerator degrees of freedom (*df*_1_) and denominator degrees of freedom (*df*_2_). In the current study, we used the digital F-table of the Excel (2010) F.INV.RT function due to its relatively easy usage and precise result. Notably, we used the F-distribution with α = 0.05, and confidence level of 95 %.

Having obtained the critical F-value, the hypothesis testing for equality of variance was carried out and in which process; the hypotheses formulated and tested were straight forward as: H_0_: $S_{x}^{2} = S_{y}^{2}$ and H_a_: $S_{x}^{2} \ne S_{y}^{2} .$

Because we placed the large sample variance in the numerator, the F-ratio, in that case, is an upper-tailed test/distribution—the null hypothesis (H_0_) is rejected or accepted when the result of F-ratio is greater than or lesser than the critical F-value respectively.

## Abbreviations

AEAs: Agricultural Extension Agents
CSIR: Council for Scientific and Industrial Research
ET: evapotranspiration
FM: frequency modulation
GDP: gross domestic product
GSS: Ghana Statistical Service
ICOUR: Irrigation Company of Upper East Region
MOFA: Ministry of Food and Agriculture
NGOs: Non-governmental Organisations
PET: potential evapotranspiration
SPSS: Statistical Package for the Social Scientists
UER: Upper East Region of Ghana
SSA: sub-Saharan Africa
FAO: Food and Agriculture Organisation of the United Nations
UNDP: United Nations Development Programme
EPA: Environmental Protection Agency
UNFCCC: United Nations Framework Convention on Climate Change
FASDEP: Food and Agriculture Sector Development Policy
AAGDS: Accelerated Agricultural Growth and Development Strategy
PSIA: poverty and social impact analysis
MDGs: millennium development goals
NEPAD: new partnership for Africa’s development
DAs: district assemblies
MWRWH: Ministry of Water Resources, Works and Housing
EAs: enumeration areas
WASCAL: West African Science Service Center on Climate Change and Adapted Land Use
ANOVA: analysis of variance
